# Synthesis and biological evaluation of titanium dioxide/thiopolyurethane composite: anticancer and antibacterial effects

**DOI:** 10.1186/s13065-024-01138-x

**Published:** 2024-02-17

**Authors:** Rana R. El Sadda, Mai S. Eissa, Rokaya K. Elafndi, Elhossein A. Moawed, Mohamed M. El-Zahed, Hoda R. Saad

**Affiliations:** 1 Chemistry Department, Faculty of Science, Damietta University, P.O. Box 34517, New Damietta, Egypt; 2 Botany and Microbiology Department, Faculty of Science, Damietta University, New Damietta, Egypt; 3 Geology Department, Faulty of Science, Damietta University, New Damietta, Egypt

**Keywords:** Titanium dioxide, Thiourea, Polyurethane foam, Ilmenite ore, MCF-7, HepG-2, Selective index

## Abstract

Nanocomposites incorporating titanium dioxide (TiO_2_) have a significant potential for various industrial and medical applications. These nanocomposites exhibit selectivity as antimicrobial and anticancer agents. Antimicrobial activity is crucial for medical uses, including applications in food processing, packaging, and surgical instruments. Additionally, these nanocomposites exhibit selectivity as anticancer agents. A stable nanocomposite as a new anticancer and antibacterial chemical was prepared by coupling titanium dioxide nanoparticles with a polyurethane foam matrix through the thiourea group. The titanium dioxide/thiopolyurethane nanocomposite (TPU/TiO_2_) was synthesized from low-cost Ilmenite ore and commercial polyurethane foam. EDX analysis was used to determine the elemental composition of the titanium dioxide (TiO_2_) matrix. TiO_2_NPs were synthesized and were characterized using TEM, XRD, IR, and UV–Vis spectra. TiO_2_NPs and TPU foam formed a novel composite. The MTT assay assessed Cisplatin and HepG-2 and MCF-7 cytotoxicity in vitro. Its IC_50_ values for HepG-2 and MCF-7 were 122.99 ± 4.07 and 201.86 ± 6.82 µg/mL, respectively. The TPU/TiO_2_ exhibits concentration-dependent cytotoxicity against MCF-7 and HepG-2 cells in vitro. The selective index was measured against both cell lines; it showed its safety against healthy cells. Agar well-diffusion exhibited good inhibition zones against *Escherichia coli* (12 mm), *Bacillus cereus* (10 mm), and *Aspergillus niger* (19 mm). TEM of TPU/TiO_2_-treated bacteria showed ultrastructure changes, including plasma membrane detachment from the cell wall, which caused lysis and bacterial death. TPU/TiO_2_ can treat cancer and inhibit microbes in dentures and other items. Also, TPU/TiO_2_ inhibits *E. coli*, *B. cereus,* and *A. niger* microbial strains.

**Supplementary Information:**

The online version contains supplementary material available at 10.1186/s13065-024-01138-x.

## Introduction

**Fig. 1:**
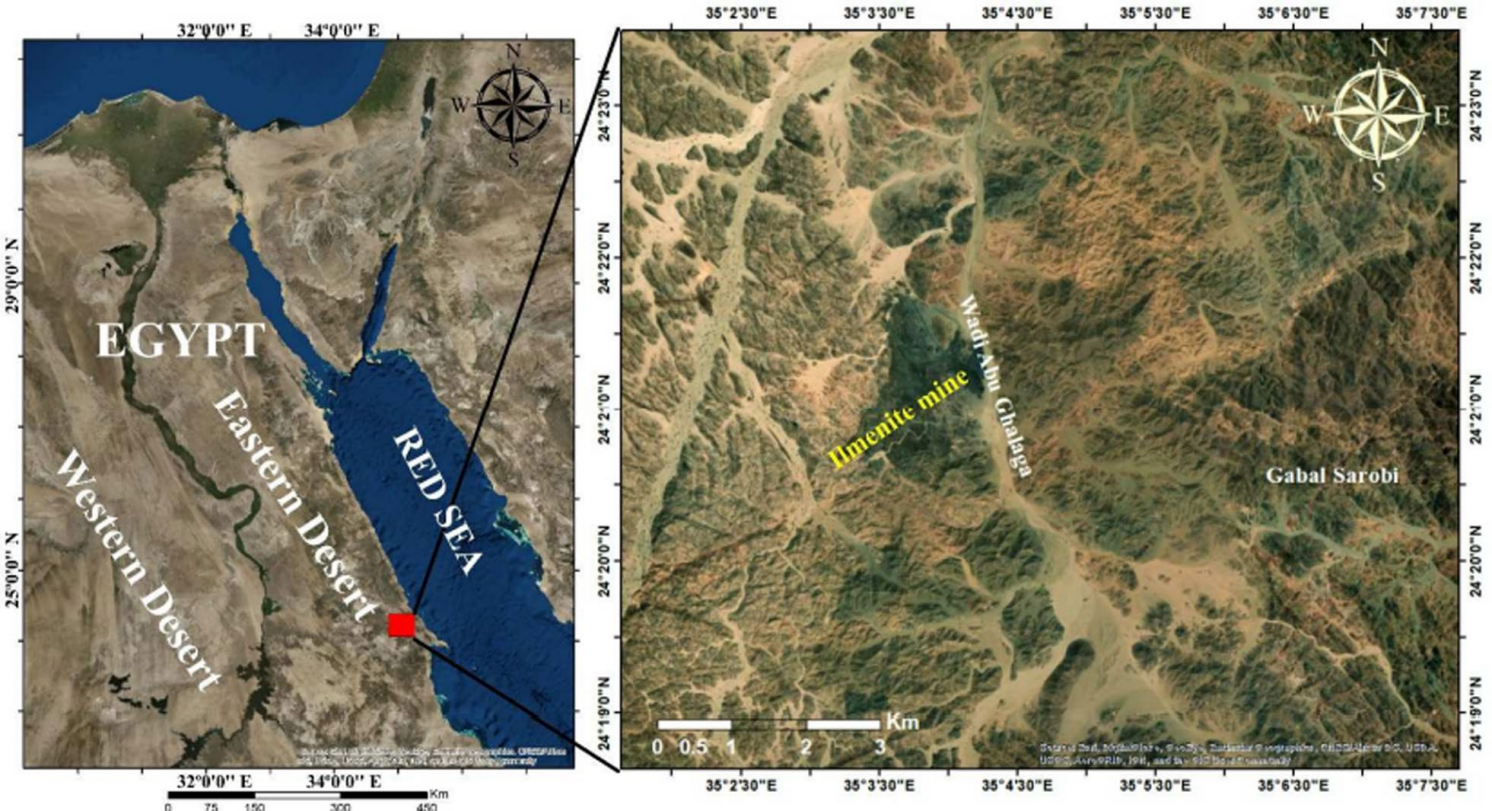
Location Map of Wadi Abu Ghalaga

Ilmenite (FeTiO_3_) is one of the most prevalent minerals and is the source of titanium dioxide (TiO_2_) [1]. It is found in the different locations of the Egyptian Eastern Desert e.g., Abu Ghalaga, Korab kanci Hamra Dome, Kolmnab Abu Dahr, Um Effein, Wadi Rahaba, Um Ginud, and Wadi El Miyah (G. El Rokham) [2–4]. There are present between 24°15ʺ and 24°25ʺ N and 35°02ʺ and 35°06ʺ E (Fig. 1). Mineralization generates bands or lenses of massive ore intercalated with gabbro layers or disseminations gradational between massive ore bands and enclosing gabbro. The main ilmenite band extends 350 m in the northwest-southwest with 50 m wide and dips 45° to the northeast. The huge ore contains 70% ilmenite, magnetite, hematite, rutile, goethite, anatase, 28% silica minerals, and 3% sulfides [5, 6].

Titanium dioxide's antibacterial properties make its use in the food sector appealing. Where, food, pharmaceuticals, and cosmetics include harmless TiO_2_. Due to their physical, chemical, and antibacterial properties, metal oxide NPs such as TiO_2_ NPs have raised concerns in recent years [7–10]. They are thermally stable and eco-friendly. Ilmenite is the principal raw material for TiO_2_ manufacturing because of the strong demand and the increasing growth of the sunscreen, toothpaste, and cosmetics sectors [11].

Polyurethane foam (PUF) is the principal polymeric substance used in industry and medicine. PUF's chemical composition and comfort are replacing earlier polymers. PUF foam resists, strengthens, and stabilizes, reducing maintenance time and cost [12–14]. Food processing, packaging, and safe transit are PUF's key uses. Refrigerators, freezers, food dryers, window frames, and furniture employ PUF for sound and cold insulation [15]. Nitrogen and sulfur give thiourea derivatives biological activity such as antibacterial, antifungal, anti-inflammatory, antioxidant, and anticancer [16, 17].

Cancer is a global killer. The WHO recommends finding new safe anticancer medicines [18]. Natural products can be exploited to generate anticancer medicines for lung, liver, breast, and pancreatic cancers [19]. Hepatocellular carcinoma (HCC) is the most frequent primary liver malignancy and the leading cancer killer worldwide [20–22]. Breast cancer kills most women worldwide, so it is necessary to focus on developing non-toxic anti-cancer medicines [18, 23].

Instead of limited chemotherapeutic treatments, stimuli-responsive delivery devices can provide tumor-targeted antitumor drugs. Drug delivery devices can reduce side effects, release, and damage to healthy tissue and organs [24]. systems (DDS) have been used to deliver therapeutic drugs for cancer treatment by either oral intake or injection [25]. Controlled drug delivery systems are improved to control the problems associated with conventional drug delivery [26]. Controlled drug delivery systems allow the drug to transport selectively to the target tissues, minimizing of its influence on vital tissues and undesirable side effects. Also, it protects the drug from rapid degradation and enhances drug concentration in target tissues, thus, lower drug doses are required [27].

In addressing the difficulties associated with conventional anticancer drugs and drug delivery systems (DDS), there is a pressing need for more focused and less side-effect-prone treatments. Researchers have explored various materials, including polymers, peptides, dendrimers, and hydrogels, as potential carriers for controlled release and targeted drug delivery. However, considerable challenges persist in the field of cancer treatment. A high molecular weight peptide, VPGVGVPGVG, was synthesized with sensitivity to temperature and pH. This peptide was employed as a carrier for the anticancer drug doxorubicin (Dox), and the conjugation was achieved through a hydrazone linkage. The specially designed peptide, the dual-sensitive peptide (DSP), demonstrated thermo-sensitive and pH-sensitive characteristics [28].

Injectable hydrogels, such as chitosan-based hydrogels, have shown potential for precise and non-invasive drug delivery [29]. Nucleic acid nanostructures have been engineered to respond to various stimuli, including pH, redox gradient, and light, for drug delivery applications [30]. Responsive delivery systems based on pH, light, and redox-cleavable polymers have been developed for controlled drug release [31]. Mesoporous silica nanoparticles (MSNs) with surface silanol groups have been utilized as versatile drug delivery platforms, with stimuli-responsive silanol conjugates enabling precise drug release [32]. Stimuli-responsive boron-based materials, such as boron nitride and boronic acid, have also been explored for controlled drug release in response to pH, light, and temperature [33]. These advancements in stimuli-responsive drug delivery systems have the potential to improve therapeutic efficacy and overcome the limitations of conventional drug delivery methods. Another study introduced the encapsulation of DHA-SBT-1214 in nanoemulsions to improve drug delivery. The properties and advantages of PEG-modified nanoemulsions, including enhanced blood circulation and efficient cellular uptake indicate that DHA-SBT-1214 delivered in nanoemulsions exhibits superior therapeutic efficacy against PPT2 cells and tumors, demonstrating potential as a novel CSC-targeted anticancer drug candidate with good tolerability in mice [34].

As a cutting-edge drug delivery technique, nanocarriers play a significant role in cancer treatment [9]. Nanoparticles, with their selective targeting capabilities and superior efficacy, have gained attraction in the field of medicine [35]. Their small size facilitates penetration through blood vessels and reduces non-specific binding, which improves their activity as a drug carrier [36]. Various targeting molecules can be conjugated on the surface of nanoparticles, which is an important aspect of drug delivery [9].

PUF, with its high surface area and biodegradation, can be used as a good substrate for cell attachment and drug delivery [37]. As an interesting substitute for conventionally utilized biodegradable polyesters, polyurethane can be employed to construct nano-carriers. Polyurethane foam is a promising choice as a targeted delivery system and a drug carrier. Polyester/ether urethanes (PURs) can replace biodegradable polyesters in nanocarriers. PURs can easily alter hydrophilic/hydrophobic equilibrium and have specific capabilities, making them a viable tailored delivery system [38].

The utilization of polymer-based micro- and nanoparticles in drug delivery systems offers distinct advantages, allowing for site-specific drug distribution within the body through cell-specific targeting. This approach provides better control over drug release kinetics [39]. Nanostructured polymers, formed by encapsulating antibiotic-loaded nanoparticles in carboxylated polyurethane, demonstrated controlled drug elution, extending antimicrobial activity for up to 8 days [40]. Alteration of the hard- and soft-segmented microstructure of polyurethane by incorporating polyester into the chain and introducing nanoparticles of polyurethane into polycaprolactone (PCL) as a carrier. This modification resulted in faster degradation, higher encapsulation efficiency, and a longer, more controlled drug release profile [41]. Coating composite shell scaffolds with gelatin-containing drug-loaded polyurethane nanoparticles is another method for maintaining scaffold microarchitecture and achieving sustained drug release [42]. Introduced a novel polyurethane nano micelle for multifunctional drug delivery with tumor-specific targeting and cleavage capabilities; this tumor-specific targeting ensures precision in drug delivery, while its cleavage capabilities offer a controlled release mechanism, potentially enhancing the therapeutic outcome while minimizing side effects [43]. Polyurethane–polyurea nanoparticles with adjusted hydrophobic and hydrophilic chains are used for drug delivery to achieve sub-30 nm nanoparticles, suggesting improved encapsulation stability compared to single-walled nanostructures [44].

Viruses, bacteria, and fungi cause most foodborne illnesses. Foodborne illnesses cause diarrhea, stomach cramps, nausea, vomiting, and fever [45, 46]. *Bacillus cereus* and *Escherichia coli* are common environmental pathogens that contaminate food. *B. cereus* is a gram-positive, motile, spore-forming bacterium, that germinates, thrives even after heat treatments, and creates enterotoxins that cause food poisoning [47]. *E. coli* is a gram-negative bacterium, which identifies fecal contamination and causes serious infections when consumed in contaminated foods. The Common food contaminant *Aspergillus niger* generates mycotoxins and aflatoxins. It also produces spores that cause aspergillosis, a dangerous lung illness [48, 49].

This work describes the TPU/TiO_2_ nanocomposite synthesis. Thiourea and TiO_2_ NP were added to PUF to increase its antibacterial, antifungal, and anticancer properties. MTT colorimetric assays assess cell viability and cytotoxicity. HepG-2 and MCF-7 cells model liver and breast cancer, respectively. *E. coli*, *B. cereus*, and *A. niger* were tested for TPU/TiO_2_ antibacterial properties as models for bacterial and fungal diseases.

## Materials and methods

### Materials

**Fig. 2:**
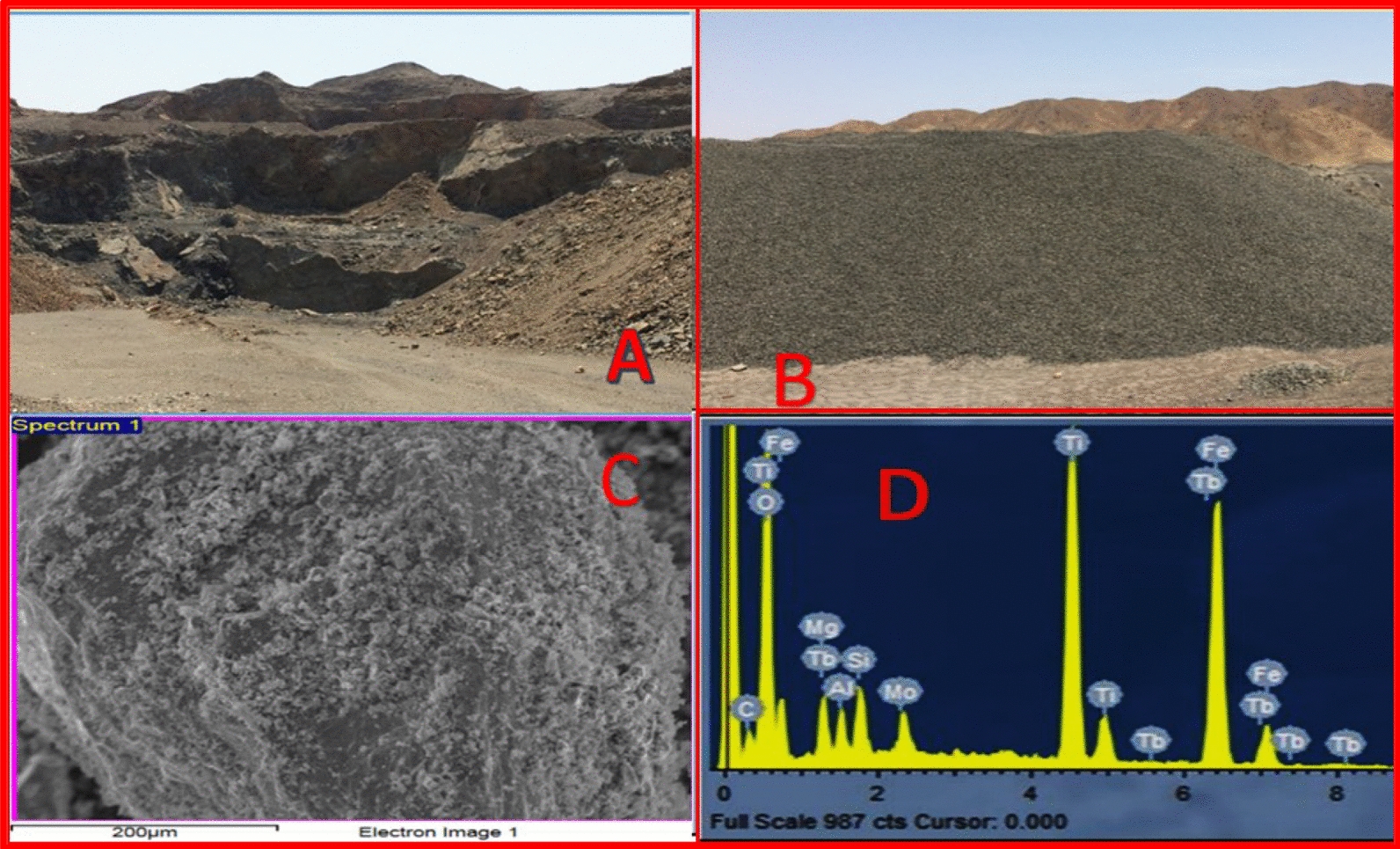
**A** Surficial oxidized ore, **B** Crushed ilmenite ore, **C** SEM image of ilmenite ore at 200 × magnification (**C**) and (**D**) EDX of ilmenite ore

TPU/TiO_2_: The ilmenite ore was obtained from the Abu Ghalaga mine, an established mining operation at the intersection of Wadi Abu Ghusun and Wadi Abu Ghalaga in the southern Eastern Desert, and identified at the field (Fig. 2A and B). The Abu Ghalaga deposit occurs on a hill overlooking Wadi Abu Galaga, 20 km west of the port of Abu Ghosun. It lies between latitudes 24°15ʺ and 24°25ʺN and longitudes 35°02ʺ and 35°06ʺE. The fresh black ore samples were pulverized to -200 mesh.

Foamex Company (Damietta, Egypt) supplied commercial open-cell flexible PUF sheets (d = 12 kg/m^3^) for foam manufacture, and sliced PUF sheets were 0.125 cm^3^ cubic. Sigma-Aldrich and Adwic (Egypt) supplied all experiment chemicals (NH_4_SCN, HCl, NaOH, NaHCO_3_, Na_2_CO_3_, benzaldehyde, ethanol 70%, methanol, acetone, and benzene).

MCF-7 and HepG-2 cells were obtained from the American Type Culture Collection (ATCC) (Rockville, MD, USA). Sigma (USA) supplied MTT, trypan blue dye, and DMSO. Lonza (Belgium) supplied fetal bovine serum, RPMI-1640, HEPES buffer, L-glutamine, gentamycin, and 0.25% trypsin–EDTA.

ATCC bacterial strains like *E. coli* (ATCC 25922) and *B. cereus* (ATCC 6633) as well as the fungal strain of *A. niger* (van Tieghem 1867) from Microbiology Laboratory, Faculty of Science, Damietta University. Bacteria and fungi were sub-cultured on nutrient broth, and Dox agar (Oxoid, UK), respectively. Pfizer Co., Ltd. supplied penicillin G and fluconazole and Sigma (USA) supplied DMF.

### Methods

The optimum conditions for the leaching of ilmenite ore and precipitated TiO_2_ were studied. The effect of acid concentration (H_2_SO_4_ of 10–70%)**,** time (14–90 min), temperature (50–250 °C), weight of ore (0.5–10 g) and acid volume (1–50 mL) were tested.

*TiO*_*2*_*NP*: 25 g of ilmenite ore was cooked at 100 °C for 2 h in 60 mL of 30% H_2_SO_4_. After cooling, it was filtrated and rinsed 3 times with dist. H_2_O. 250 mL dist. The filtrate was heated at 90 °C for 3 h with H_2_O. The residue was filtrated, washed, dried overnight, and calcined at 800 °C for 2 h to yield TiO_2_NP [50–52].

*TPU*: 10 g of PUF cubes were heated with stirring in 1 mol/L HCl for 3 h and rinsed well with distilled water. PUF cubes were washed in 50 mL of concentrated HCl and then 25 mL of 5 g/l NH_4_SCN was added [53].

*TPU/TiO*_*2*_: 4 g TPU and 2 g TiO_2_ were refluxed in 200 mL ethanol at 60 °C for 2 h. TPU/TiO_2_ was rinsed with distilled water, and ethanol, then it was air-dried.

### Characterization

The infrared (IR) spectra were carried out using a KBr disc (KBr pellet) on a JASCO FTIR-410 spectrometer (Germany) in the 4000–400 cm^−1^ region. UV/VIS JASCO Spectrometer V-630 (Japan) was used for absorbance measurements. It measures the amount of light absorbed by a sample through a reference sample (water is the blank). MINIFLIX Benchtop Powder X-ray Diffractometer (USA) identified the crystalline phase. XRD data was performed in the faculty of sciences at Banha University. The morphological characteristics and elemental composition of the ilmenite sample were characterized using SEM and EDX (JEOL model JSM-6510LV, USA), at an accelerated voltage of 20 kV in the secondary electron mode. The fracture surface was vacuum-coated with gold and examined at 200 µm magnification. The size and morphology of the prepared TiO_2_NPs were examined using a transmission electron microscope (TEM) Model Talos TM 120C, Thermo Fisher, Scientific, UK with an acceleration voltage of 120 kV. TEM was carried out in the Electron Microscope Unit at Damietta University. The ultrastructure study of treated bacteria was investigated using a JEOL JEM-2100, Japan, Electron microscope unit, Mansoura University operated at an accelerating voltage of 200 kV. The cells were cross-sectioned using an ultra-microtome, stained, and examined using TEM on carbon-coated copper grids (Type G 200, 3.05 μM diameter, TAAP, U.S.A.). Zeta potential of TPU/TiO_2_ dispersion in water was conducted using Zetasizer, Malvern Instruments, Nano-ZS. It was measured three times at room temperature.

The acidic and basic sites of TPU/TiO_2_ were determined using Boehm’s titration. 0.5 gm of TPU/TiO_2_ was added to 10 mL of 0.05 mol/L NaHCO_3_, Na_2_CO_3_, NaOH, and HCl. After 24 h of soaking, the solutions were titrated against 0.05 mol/L HCl and NaOH. The total acidity (the sum of carboxyl, lactone, and phenolic groups) was recorded. The surface charge of TPU/TiO_2_ was evaluated over the initial pH range of 2–14 and pH at the zero-charge point (pH_PZC_) was determined. 0.5 gm of TPU/TiO_2_ was added to 10 ml of each buffer solution and after 24 h, the final pH was also measured. The differences between the initial and final pH values were plotted against the initial pH. The chemical stability of TPU/TiO_2_ was tested in different buffer solutions (pH: 2–14) and different organic solvents (e.g., CH_3_OH, CH_3_COCH_3_, C_6_H_6_, C_6_H_5_CH3, DMF, and DMSO). 0.5 g of TPU/TiO_2_ was soaked in 10 mL of each buffer solution and organic solvent for 24 h, then filtrated, dried, and weighted.

### Anticancer activity

The Regional Centre for Mycology and Biotechnology (Al-Azhar University, Cairo) cultivated MCF-7 and HepG-2 cells. RPMI-1640 medium with 10% inactivated fetal calf serum and 50 µg/mL gentamycin supported cell growth. Cells were subcultured two to three times a week at 37 °C in a humidified environment with 5% CO_2_.

*Cytotoxicity*: Colorimetric MTT assays assessed TPU/TiO_2_'s cytotoxicity on two cancer cell lines (breast-cancer cell line MCF-7 and hepatocellular carcinoma cell line HepG-2) cells in addition to healthy mammalian cells from African Green Monkey Kidney (Vero). MCF-7 and HepG-2 cells were suspended in media (5 × 10^4^cell/well) in 96-well tissue culture plates for 24 h. Twelve TPU/TiO_2_ concentrations were introduced in three duplicates. Each 96-well plate had 6 vehicle controls with medium or 0.5% DMSO. The MTT test counted live cells after 24 h. Briefly, the media was withdrawn from the 96 well plates and replaced with 100 µL of fresh culture RPMI 1640 medium without phenol red, followed by 10 µL of the 12 mM MTT stock solution (5 mg MTT in 1 mL PBS) in each well, including the untreated controls. 96 well plates were incubated at 37 °C and 5% CO_2_ for 4 h. An 85 µL aliquot of the medium was withdrawn from each well, and 50 µL of DMSO was added and mixed thoroughly with the pipette and incubated at 37 °C for 10 min [54, 55]. To count live cells, 590 nm optical density was assessed, calculating cell viability (Additional file 1). $Viability = (ODt/ODc)\times 100\%$ where OD_t_ is the mean optical density of TPU/TiO_2_-treated cells and OD_c_ is that of untreated cells. The survival curve of each cancer cell line following TPU/TiO_2_ treatment was plotted as a function of drug concentration and surviving cells. From graphic plots of the dose–response curve for each concentration, the 50% inhibitory concentration (IC_50_) was determined by the following equation estimated the half-maximal effective concentration (EC_50_) of TPU/TiO_2_, which yields half-maximum response: $Y=Min+ \frac{Max -Min}{1+ {(\frac{X}{EC50})}^{Hill coefficient}}$

### Antimicrobial action

*Agar well-diffusion method*: TPU/TiO_2_'s antimicrobial activity was tested against gram-negative *E. coli*; gram-positive *B. cereus*; *A. niger*. Clinical and Laboratory Standards Institute recommendations were followed for agar well-diffusion [56]. Nutrient and Dox agar were autoclaved (121 °C, 15 min) and coaled at 47 °C. Each 100 µL microbial culture (1–2 × 10^8^ CFU/mL) was injected into the agar media. Triplicate sterile Petri dishes were filled with inoculated agar material. After solidification, sterilized corkborers punctured 5-mm wells. TPU/TiO_2_, Penicillin (antibacterial), and Fluconazole (antifungal) aliquots of 300 µg/mL in DMF were applied to the wells separately. Inoculated nutritional agar plates were incubated at 37 °C for 24 h and Dox agar plates at 30 °C for 5 days. After incubation, mm-sized inhibitory zones were detected.

*Minimum inhibitory concentration (MIC)*: The TPU/TiO_2_'s MIC against gram-negative bacteria *E. coli* and gram-positive bacteria *B. cereus* was investigated [57]. Nutrient broth was made, autoclaved at 121 °C for 15 min, and coaled at 47 °C. In two sets of flasks, 100 μL of *E. coli* and *B. cereus* (0.5 McFarland standards (1–2 × 10^8^ CFU/mL)) were inoculated. Various quantities of sorbent (0–1000 μg/mL) were carefully put into each flask, with one serving as a positive control to monitor the normal development of the microbial cells in the absence of TPU/TiO_2_. A negative control flask containing only cells and DMF was also made. For 24 h, the flasks were incubated in a shaker incubator (100 rpm) at 37 °C. Turbidity or cloudiness of the broth indicates the development of the inoculums in the broth, and the lowest concentration of TPU/TiO_2_ that inhibited the growth of the test organism was chosen as the MIC. The optical density (OD) at 600 nm was measured spectrophotometrically to calculate the MIC value. The following formula was used to compute the growth inhibition percentage:

$$\text{Growth}\; \text{inhibition} \%=\left[\left({OD}_{C}-{OD}_{t}\right)/{OD}_{C}\right]\times 100$$

where ODc and ODt are the OD of the control (without TPU/TiO_2_) and tested TPU/TiO_2_, respectively.

*Minimum microbicidal concentration (MBC)*: Flasks of MIC for TPU/TiO_2_ that had no apparent bacterial growth were inoculated into nutrient agar plates using the pour plate method and then incubated at 37 °C for 24 h. The MBC values of the antibacterial agents were determined with no apparent colonial bacterial growth plates El-Fallal [58].

*Ultrastructural study*: TPU/TiO_2_ was tested on microbial ultrastructure using *E. coli.* Bacterial cell cultures were treated with TPU/TiO_2_ for 2 h at 37 °C in nutritional broth. Bacteria were centrifuged at 5000 rpm for 15 min, and treated with 2.5% glutaraldehyde and 0.1 M cacodylate buffer, pH 7. TEM studied the ultrastructure of untreated and TPU/TiO_2_-treated bacteria.

*Data analysis*: The mean ± standard deviation (S.D.) is the standard error of the mean. GraphPad Prism 6, San Diego, CA, estimated IC_50_. Experimental data were analyzed using SPSS 19.0.

## Results and discussion

### Ilmenite and TiO_2_ NP characterization

Ilmenite ore morphology and surface structure were examined using SEM at 200 × magnification (Fig. 2C). The particle size of ilmenite was big and rough. EDX was performed to determine the elemental composition of ilmenite ore (Fig. 2D). The weight percentages of the principal components in the ilmenite matrix were carbon (9.5%), oxygen (39.3%), titanium (15.1%), iron (24.0%), terbium (4.7%), molybdenum (2.6%), magnesium (1.8%), silicon (1.7%), and aluminum (1.3%).

Tabassi et al. synthesized TiFe_2_O_4_@Ag NPs which exhibited spherical shapes with a size range of 20–60 nm, and excellent stability (zeta potential of -47.7 mV). Anticancer evaluations demonstrated significant toxicity of TiFe_2_O_4_@Ag NPs toward AGS gastric cancer cells (IC_50_ = 69.6 µg/mL) compared to normal HEK293 cells (IC_50_ = 130 µg/mL) through the MTT assay. This study introduces TiFe_2_O_4_@Ag NPs as a novel and promising anticancer agent, emphasizing the need for further characterization for potential biomedical applications [59]. In another study by Cobos et al., they synthesized silver nanoparticles that were incorporated AgNPs into polymer matrices, such as polyvinyl alcohol (PVA) or chitosan, to create nanocomposites with enhanced antibacterial activity. These nanocomposites have shown effectiveness against various bacterial strains. Additionally, they can be functionalized with anticancer drugs for combined antibacterial and anticancer applications [60].

**Fig. 3:**
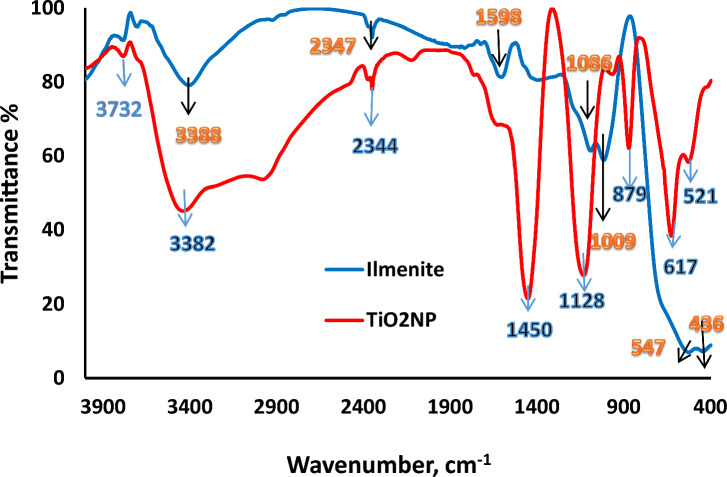
FTIR spectra for ilmenite ore and titanium dioxide nanoparticles

FTIR identified the ilmenite ore's functional groups (Fig. 3). OH group stretching caused ilmenite's 3388 cm^−1^ absorption band. Carbon dioxide O=C=O stretching caused 2347 cm^−1^ bands. OH bending bands are at 1598 cm^−1^. The Ti–O matrix included 1086, 1009, 547, and 436 cm^−1^ peaks.

A UV–visible spectrophotometer at room temperature measured the ilmenite diffuse reflectance spectrum from 200–900 nm. The ilmenite sample strongly absorbs UV light at 234, 292, and 357 nm. Leaching mineral oxides from ilmenite ore with 30% H_2_SO_4_, precipitating with water, and calcining at 800 °C yielded TiO_2_ NP. The FTIR spectra of TiO_2_NP showed that the three stretching peaks appeared at 3732, 3382, and 2344 cm^−1^ for OH, H_2_O, and O=C=O groups (Fig. 3). These groups were due to the physically and chemically adsorbed on the surface of TiO_2_NPs. The TiO_2_NP skeleton is represented by 1450 cm^−1^ bands. The Ti–O and Ti–O–Ti matrix had strong peaks at 1128, 879, 617, and 521 cm^−1^.

**Fig. 4:**
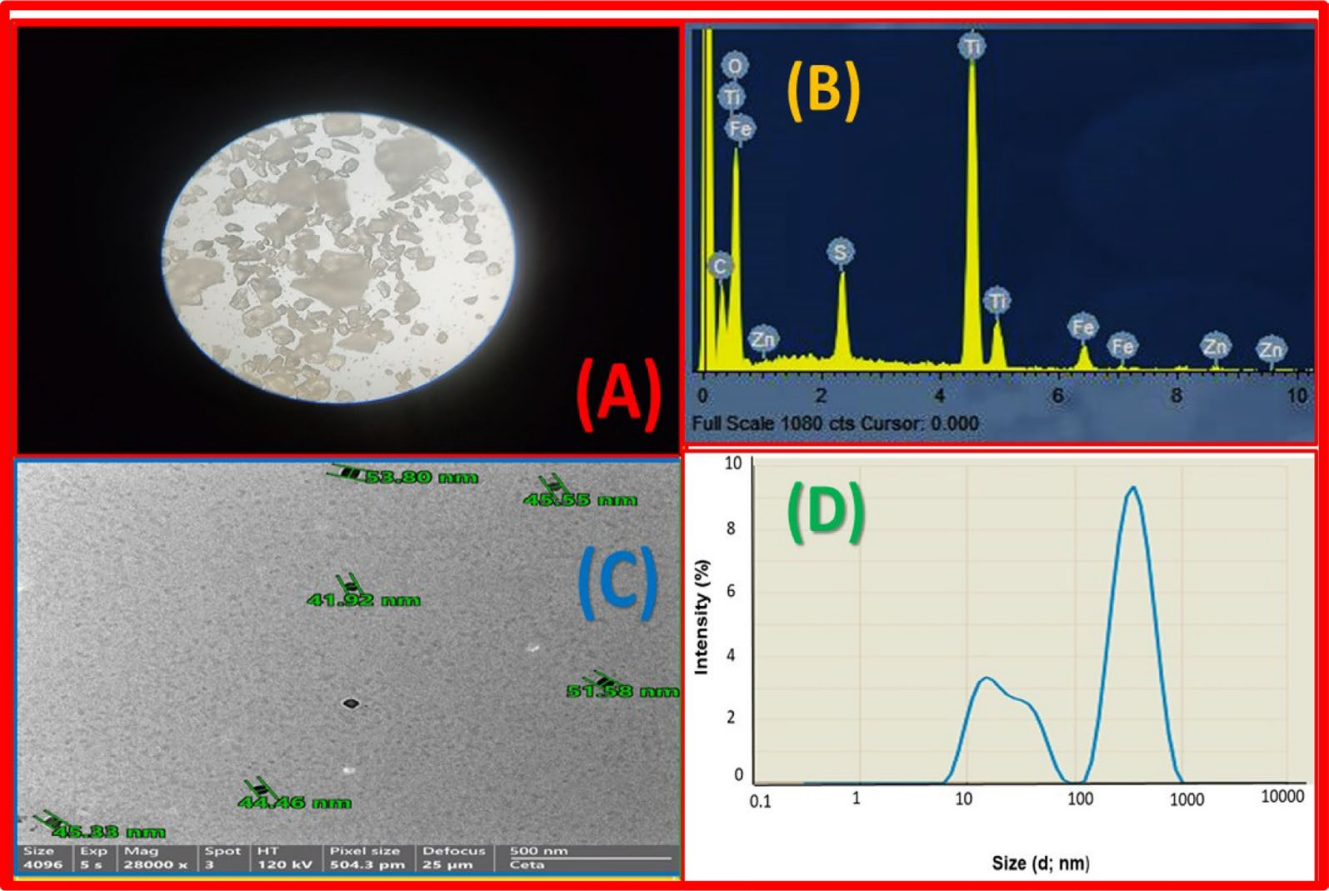
**A** White color image of TiO_2_NPs, **B** EDX of TiO_2_NPs **C** TEM of TiO_2_NPs and **D** TiO_2_NPs particles size distribution by Intensity

Due to its white color, TiO_2_NP was utilized industrially as a white pigment in paper, ceramics, rubber, textiles, paints, inks, cosmetics, and food coloring. Figure 4A shows the dazzling white TiO_2_NP from ilmenite ore. The weight percentages of the key elements in TiO_2_NP naturally synthesized from the ilmenite ore sample were O (52.2%), Ti (23.4%), Fe (2.8%), C (17.6%), S (3.1%), and Zn (0.9%), which determined the EDX spectrum (Fig. 4B).

TEM images showed TiO_2_NP form and size (Fig. 4C). Single-particle TiO_2_NP has uneven diameters. TiO_2_NP particles averaged 47.1 nm. This particle size matched the literature results.

The TiO_2_NP electronic spectrum was 200–900 nm. TiO_2_NP absorbed UV light at 304 and 349 nm. From reflectance spectra, the optical bandgap (Eg) of TiO_2_NP was calculated: where C is a constant, α is the absorption coefficient, A is the absorbance, and t is the thickness. The wavelength (nm) and energy h (eV) were computed. The (αh)^2^ plotted versus h gives the energy gap at h = 0. TiO_2_NP's direct band gap, semiconductor energy was 3.5 eV.

### TPU/TiO_2_ characterization

The particle size distribution of the TPU/TiO_2_ nanocomposite varied from 41.58 to 63.94 nm (Fig. 4D). The mean diameter of the nanoparticles, according to DLS, was found to be 50.1 ± 12.09 nm.

**Fig. 5:**
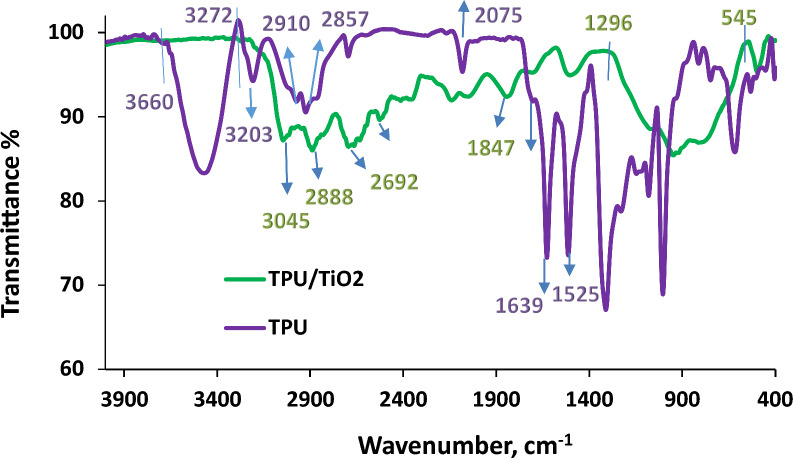
FTIR spectra for TPU and TPU/TiO_2_ nanocomposite

FTIR spectroscopy identified TPU and TPU/TiO_2_ functional groups (Fig. 5). In the FTIR spectrum of TPU, the characteristic peaks of TPU appeared at 2075 cm^−1^ (N=C=S), and 1540 cm^−1^ (N=C). Broadband from 3660 to 3272 cm^–1^ (–NH and –OH), several sharp peaks at 2910, 2867, 2857 cm^−1^ (–CH), 1668 cm^−1^ (C–O), 1639 cm^−1^ (C=C), and 1525 cm^−1^ (C–O–C) characterize the matrix of PUF. In the FTIR spectrum of TPU/TiO_2_, TPU peaks were found at 3045, 2888, 2692, 2528, 1820, 1680, 2146, 2048, 1847, and 1076 cm^−1^. Ti–O's characteristic band changed to 1296–545 and peaked at 487 cm^−1^.

**Fig. 6:**
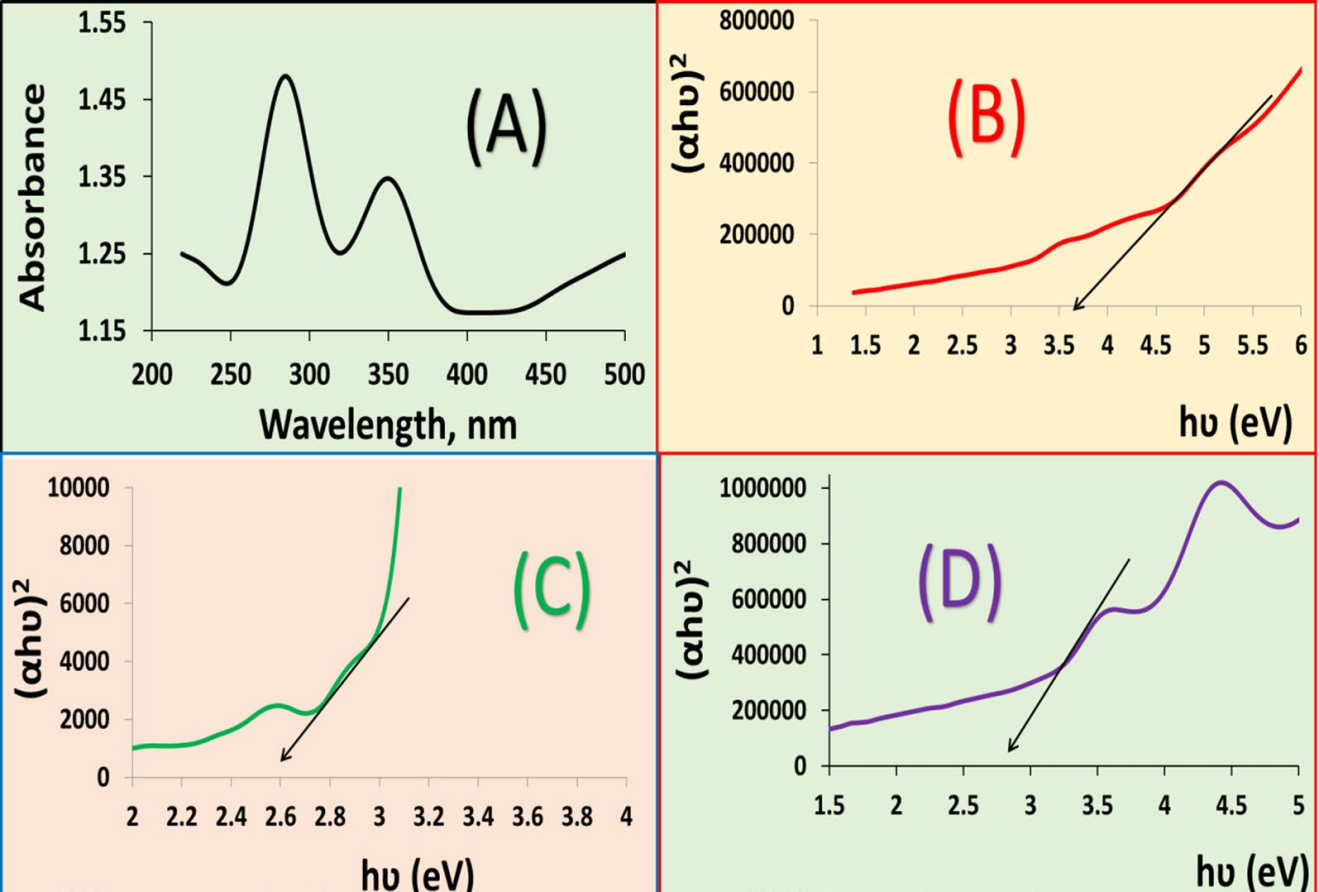
**A** UV/Vis spectra for TPU/TiO_2_, **B** band gap energy of TiO_2_, **C** band gap energy of TPU and **D** band gap energy of TPU/TiO_2_

TPU and TPU/TiO_2_ UV–Vis spectra were measured at 200–900. TPU substantially absorbed UV radiation at 350 nm in water, while TPU/TiO_2_ absorbed it at 284 and 350 nm. For a straight band gap semiconductor, TPU and TPU/TiO_2_ have 2.5 and 2.7 eV band gaps, respectively (Fig. 6). TPU/TiO_2_'s greater particle size explains its lower energy gap (2.7 eV) than TiO_2_NP's (3.5 eV). TPU/TiO_2_ has higher surface polarity and electrical conductivity than TiO_2_NP.

The TPU/TiO_2_ and TPU magnetic susceptibility were determined using Evans balance data using the following equation: ${\chi }_{g}=CL (R-{R}_{0})/{10}^{9}(M-{M}_{o})$*.* Where: C is the balance calibration constant (1.35 cm), L is the sample height in cm, R is the balance reading for the sample in a tube, R_o_ is the empty tube reading, M is the sample mass and tube in g, and M_o_ is the empty tube mass in g.

TPU/TiO_2_ has a low but positive magnetic susceptibility of 1.75 × 10^–6^ cm^3^/mol. Paramagnetic TPU/TiO_2_ was weakly magnetic. TPU has a diamagnetic magnetic susceptibility of 0.32 × 10^–6^ cm^3^/mol.

**Fig. 7:**
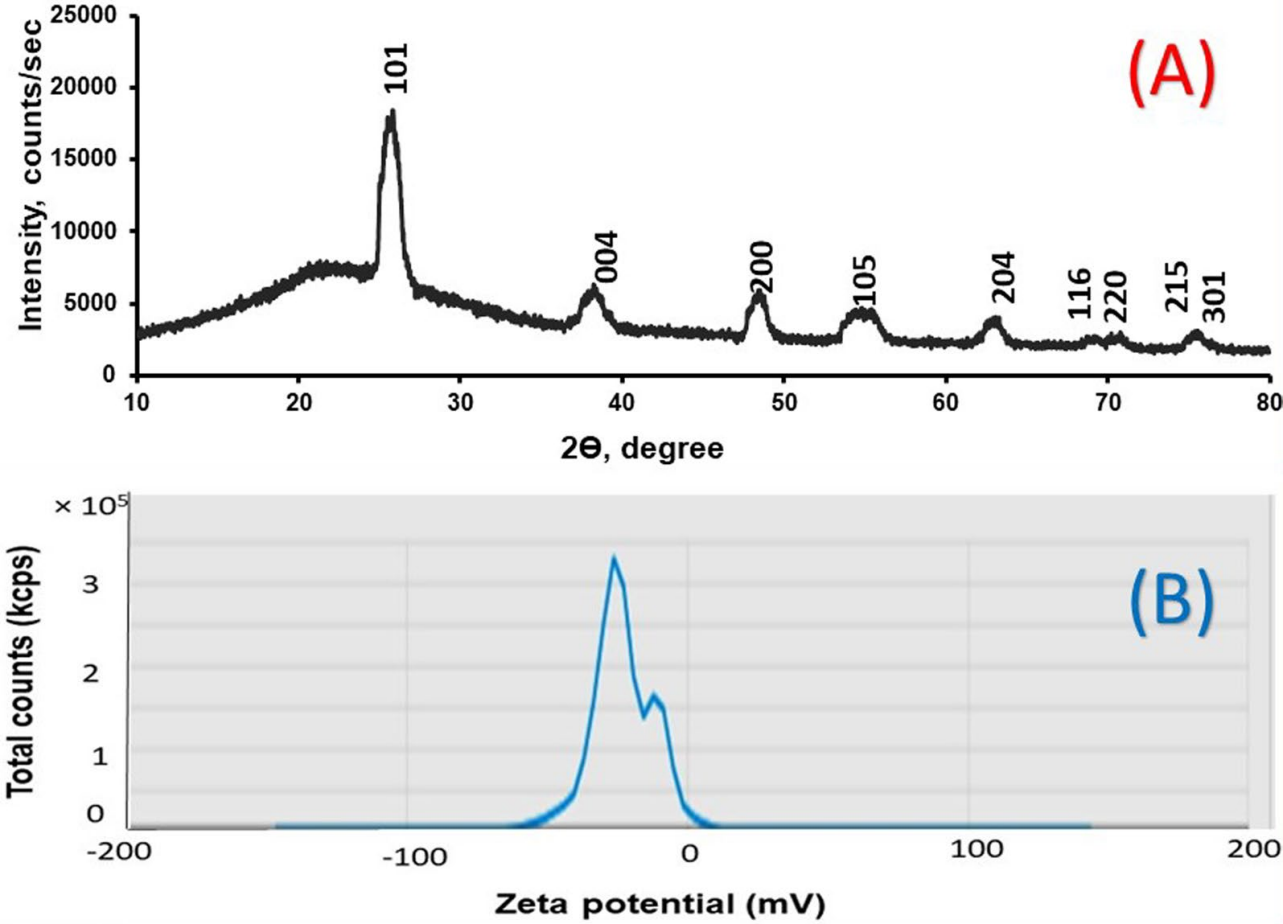
**A** XRD patterns of TPU/TiO_2_ nanocomposite and its corresponding Phase names **B** Zeta Potential Distribution of TPU/TiO_2_

The X-ray diffraction patterns of TPU/TiO_2_ are shown in Fig. 7A and the peak details are in Table 1. The XRD patterns of TPU/TiO_2_ showed a strong and narrow diffraction peak, which refers to the good crystallinity of the TPU/TiO_2_ nanocomposite. The characteristic diffraction patterns of TPU/TiO_2_ appeared at 2Ɵ = 25.7, 38.2, 48.4, 54.8, 62.9, 69.1, 70.7, 75.4 and 76.5; these correspond to crystal planes of (101), (004), (200), (105), (204), (116), (220), (215) and (301). The diffraction peaks at 25.7 (101), 38.2 (004), 48.4 (200), 54.8 (105), 62.9 (204), 70.7 (220) and 75.4° (215); were attributed to the anatase phase of TiO_2_NPs. These patterns agree with the Joint Committee on Powder Diffraction Standards (JCPDS card no. 21-1272). However, the diffraction peaks at 2θ around 69.2 (116) and 76.5° (301); indicating the coupling between TPU and TiO_2_NPs. The TPU/TiO_2_ X-ray pattern exhibits a tetragonal crystal system (space group 141: I41/ amd:2). The order of their lattice parameters is as follows: (a = b = 0.38 nm, c = 0.95 nm and α = β = γ = 90°). The results showed that modification of TPU with TiO_2_NPs caused a phase change in the crystal structure. The particle size (D) of TPU/TiO_2_ was calculated using the Debye–Scherrer equation ($D=0.9\lambda /\beta cos\theta$). Where λ is the wavelength of X-ray (0.15418 nm), β is the peak full width at half maximum (FWHM) in radians and θ is the Bragg diffraction angle. The average crystallite size of TPU/TiO_2_ was in the range of 4.6–33.14 nm. Inter-planar spacing between atoms (d-spacing) is calculated using Bragg’s Law: $2d sin\theta = n\lambda$.

**Table 1:** XRD data for TPU/TiO_2_

No.	2θ, °	Size, nm	FWHM, °	d, nm	Phase name
1	25.09881	18.797	0.4522	0.354516	Unknown
2	25.70177	8.68939	0.9794	0.346334	1 0 1
3	38.20488	5.6521	1.5536	0.235379	0 0 4
4	48.36408	8.40006	1.0828	0.188044	2 0 0
5	54.77334	4.6054	2.0291	0.167458	1 0 5
6	62.91277	8.41529	1.1558	0.147609	2 0 4
7	69.15261	10.36716	0.972	0.135734	1 1 6
8	70.70968	8.64852	1.1763	0.133122	2 2 0
9	75.42829	9.49174	1.105	0.125923	2 1 5
10	76.55398	33.14299	0.3189	0.124349	3 0 1

Zeta potential analysis was performed to study the surface stability and charge of the nanoparticles. The zeta potential analysis revealed that the nanoparticles were stable with a zeta potential value of − 22.85 ± 10.81mv Fig. 7B. It was stated that TPU/TiO_2_ nanoparticles had negatively charged on their surface.

Boehm's titration determined TPU/TiO_2_'s acidic and basic sites. TPU/TiO_2_ had 60 mmol/g acidic sites and minimal basic sites. Over an initial pH range of 2–14, the surface charge and pH at zero charge point (pH_PZC_) of TPU/TiO_2_ were determined. TPU/TiO_2_ has a pH_PZC_ of 4. The TPU/TiO_2_ surfaces are positively charged at pH < 4 and negatively charged at pH > 4. The chemical stability of TPU/TiO_2_ was examined in several buffer solutions (pH 2–14) and organic solvents e.g., CH_3_OH, CH_3_COCH_3_, C_6_H_6_, C_6_H_5_CH_3_, DMF, and DMSO. TPU/TiO_2_ weights were unaffected by testing solutions and solvents, after 24 h soaking, confirming their chemical stability.

### TPU/TiO_2_ antitumor activity

**Fig. 8:**
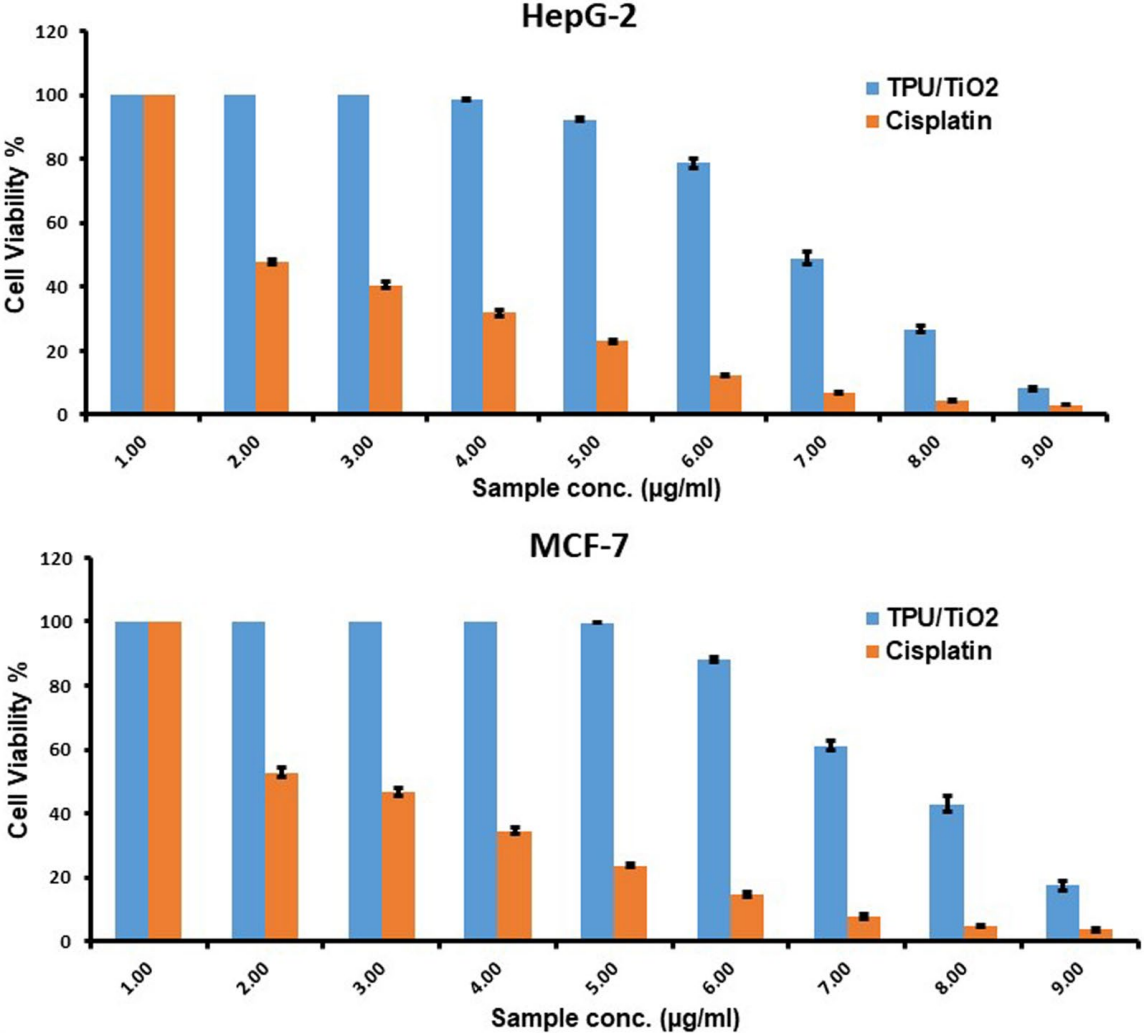
**A** Cytotoxicity assessment of TPU/TiO_2_ and Cisplatin (reference drug) against MCF-7 cells after 24 h of treatment **B** Cytotoxicity assessment of TPU/TiO_2_ and Cisplatin (reference drug) against HepG-2 cells after 24 h of treatment

**Fig. 9:**
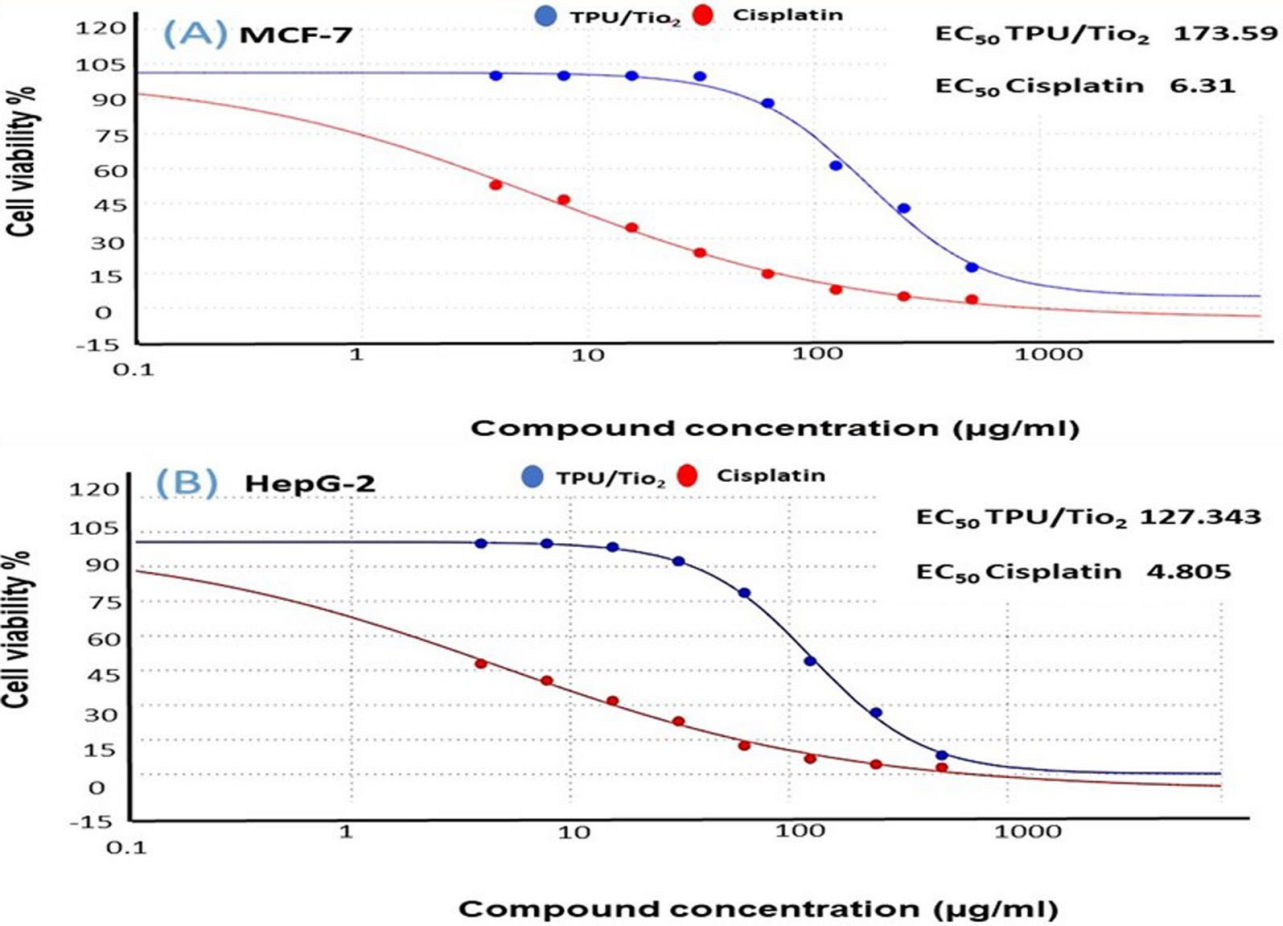
Semi Log dose–response curves and calculated EC_50_ values of TPU/TiO_2_ (in blue) in comparison to Cisplatin (in red) against (**A**) MCF-7 (**B**) HepG-2 cells. Cell viability was determined using MTT assay

TPU/TiO_2_ was tested for cytotoxicity on MCF-7 and HepG-2 cells (Fig. 8). MCF-7 and HepG-2 cell viability curves were estimated after 24 h of treatment with TPU/TiO_2_ (0–500 μg/mL). TPU/TiO_2_ was cytotoxic to HepG-2 and MCF-7 cells with IC_50_ values of 122.99 ± 4.07 and 173.58 ± 6.82 µg/mL respectively. Cisplatin IC_50_ values for HepG-2 and MCF-7 cells were 3.58 ± 0.34 and 5.72 ± 0.59 µg/mL respectively (Table 2). TPU/TiO_2_ has a lower IC_50_ for HepG-2 than MCF-7, indicating that TPU/TiO_2_ will work better for HepG-2 than MCF-7. Nanocomposite specifically reacts to the tumor microenvironment, enhancing drug accumulation in the tumor while reducing its side effects on non-cancerous tissues, which improves therapy. EC_50_ for HepG-2 and Cisplatin were 127.34 and 4.81, respectively. Moreover, EC_50_ for MCF-7 and cisplatin were 173.59 and 6.31 (Fig. 9 and Table 3). TPU/TiO_2_ nanocomposite had greater IC_50_ and EC_50_ values than cisplatin. Toxicity, pharmacokinetics, and side effects should be considered when comparing drugs. A novel drug with a higher IC_50_ and high selective index (SI) value may be as effective as cisplatin. The calculated SI ratios of TPU/TiO_2_ for MCF-7 and HepG-2 were 0.729 and 1.029, respectively. While SI values of cisplatin for MCF-7 and HepG-2 were 0.360 and 0.575, respectively. This is further evidence indicating that applying TPU/TiO_2_, contrary to cisplatin, for treating cancer cell lines is expected to show minimal or no toxicity for healthy cells. Furthermore, TPU/TiO_2_ shows more activity against HepG-2 than MCF-2. Our results agreed with those of Hassan et al., who used PdNPs and polyionic cross-linked chitosan (PICCS@Pd) nanocomposite as a nanocarrier for the delivery of doxorubicin (DOX) and 5-fluorouracil (5-FU) individually and in a cocktail. This new nanocomposite demonstrated excellent selectivity to attack tumor cells (MCF-7 and HT-29) compared to normal cells (HSFs) [61]. El Sadda et al. studied vitamin C and aspirin against the HepG-2 cell line; they showed higher selectivity than doxorubicin, which is referred to as standard therapy [62]. Also, similar to Payolla et al., vanadium-based compounds showed a greater selectivity index and better in vitro results than cisplatin [63].

**Table 2:** IC_50_ and selectivity index SI values of TPU/TiO_2_ and Cisplatin in MCF-7 and HepG-2 cells (24 h treatment)

Cell line	IC_50_ values ± SD (µg/ml) after 24 h			Selective index (SI) = IC50 (VERO) /IC50 (Carcinogenic cell line)	
	MCF-7	HepG-2	Vero	MCF-7	HepG-2
TPU/TiO_2_	173.58 ± 6.82	122.99 ± 4.07	126.61 ± 3.89	0.729	1.029
Cisplatin	5.72 ± 0.59	3.58 ± 0.34	2.06 ± 1.58	0.360	0.575

MCF-7: Breast cancer cell line. HepG-2: Hepatocellular carcinoma. Vero: Mammalian cells from African Green Monkey Kidney.Selectivity index (SI) = [IC50 (vero)/IC50 (HePG2)]. The higher the SI ratio, the theoretically more effective and safer a drug

**Table 3:** EC_50_ of TPU /TiO_2_ and Cisplatin in MCF-7 and HepG-2 cells

Cell line	EC50	
	TPU/TiO_2_	Cisplatin
MCF-7	173.59	6.31
HepG-2	127.34	4.81

Gambogic acid-TiO_2_ nanocomposite caused photodynamic therapy (PDT)-induced apoptosis and necrosis in HepG-2 cells, according to another study. TiO_2_ nanofibers can reduce drug intake in HepG-2 cells and prevent adverse effects on normal cells and tissue, which could be used in cancer treatment alliances. These nanocomposites modulate medication release [24]. Thiourea derivatives inhibited PTKs, topoisomerase II, human-type proteins, and DNA repair production, making them attractive anticancer treatments. Abbas et al. found that thiourea that used it as a structure modification selectively killed HepG-2 cancer cells over MCF-7 [64]. We should say that the incorporation of nano-sized carbon into the polyurethane matrix improved the antithrombogenicity of the polyurethane materials. It might be a novel and promising approach to developing biomaterials with high blood compatibility.

### Antimicrobial TPU/TiO_2_

While polyurethane foam (PUF) used for industrial and medical uses does not have to be antiseptic, antimicrobial activity is essential for medical indications such as food processing, packaging, and surgical instruments So, is PUF's antibacterial action related to any negative alterations in cell viability in vitro? To answer this question, we investigated the antimicrobial cytotoxic activity properties of a titanium dioxide/the polyurethane composite (TPU/TiO_2_). TPU/TiO_2_ represented low cytotoxic and high antimicrobial activities. In contrast, Cisplatin is known to have high cytotoxic and antimicrobial activity. Although PUF used for industrial and medical purposes does not have to be antimicrobial, the control of infection transmission from one person to another as well as the need for new effective agents against microbial resistance and low cytotoxic anticancer agents require antimicrobial activity.

#### Agar well diffusion

**Fig. 10:**
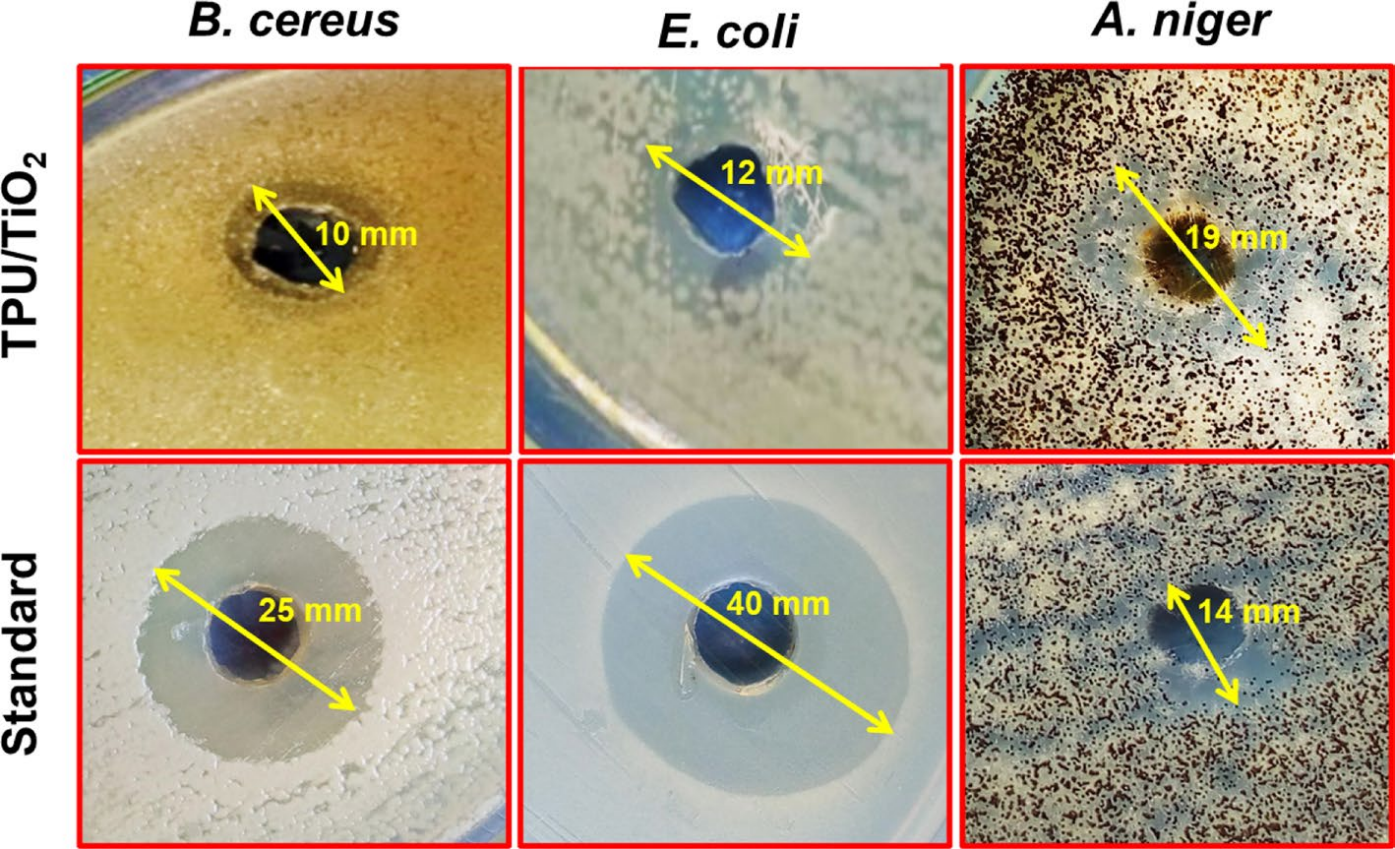
Antimicrobial activity of TPU/TiO_2_ in comparison with Penicillin (standard antibacterial) and Fluconazole (standard antifungal) using agar well diffusion method against *B. cereus*, *E. coli*, and *A. niger*. Arrows denote the diameter of inhibition zones (mm)

TPU/TiO_2_ inhibited *E. coli*, *B. cereus*, and* A. niger* using agar well diffusion. Figure 10 and Table 4 show that TPU/TiO_2_ has good antibacterial action. TPU/TiO_2_ inhibited gram-negative *E. coli* better. Gram-positive *B. cereus* with 12- and 10-mm inhibitory zones. Penicillin G outperformed TPU/TiO_2_. The TPU/TiO_2_ was more antifungal against *A. niger* than Fluconazole. Benzyl penicillin or penicillin G (the used antibiotic) generally has poor gram-negative activity, on the other hand, if it was dissolved in an organic solvent such as DMF which had synergistic effects, via damage to the bacterial membranes, that increased its potency by providing greater access to the periplasm/peptidoglycan. Literature value ranges for minimum inhibition concentration (MIC) of penicillin G against *E.* coli (ATCC 25922) were recorded as 16–64 µg/mL and inhibition zones ranged from 25–45 mm [65–70]. While Hossain et al. described *E. coli* (ATCC 25922) as an intermediate-resistant bacteria that had a MIC of penicillin G reaching 16 μg/mL [71]. TPU/TiO_2_ was more antifungal against *A. niger* than Fluconazole. Acidic substances like peptidoglycan give microorganisms a negative charge. Gram-positive bacteria have higher cell wall negative charge values due to larger peptidoglycan coatings [72, 73]. TPU/TiO_2_'s negative charge may repel germs, reducing its antibacterial efficacy compared to Penicillin G. Gram-positive bacteria (high peptidoglycan content) had a greater repulsion force than gram-negative bacteria, which may reduce their antibacterial activity.

**Table 4:** Antimicrobial effect of TPU/TiO_2_ in comparison with Penicillin (standard antibacterial) and Fluconazole (standard antifungal)

Compound	Zones of inhibition (mm ± SD)		
	*E. coli*	*B. cereus*	*A. niger*
TPU/TiO_2_	12 ± 0.06	10 ± 0.03	19 ± 0.14
Penicillin	40 ± 0	25 ± 0.03	–
Fluconazole	–	–	14 ± 0.14

Despite ROS production and membrane damage, TiO_2_NPs may be effective antibacterial agents against microbial pathogens [74]. Thiourea is bound to peptides in the microbial cell wall, substantially down-regulating glucose, alanine, aspartic acid, glutamic acid, arginine, and proline metabolism. This action may kill enzymes that are crucial to microbial development [75]. Marzi et al. proposed thiourea derivatives as potent killers for *B. cereus* and *E. coli* that produced inhibition zones of 11 and 9 mm, respectively [76]. Thiourea compounds also inhibited *E. coli* with inhibition zones ranging from 14 to 23 mm according to [77]. Anbumani et al. also found that TiO_2_ NPs kill *B. subtilis* (15 ± 0.46 mm), *E. coli* (35 ± 0.44 mm) *and A. niger (*21 ± 0.46 mm) [78].

#### MIC and MBC studies

**Fig. 11:**
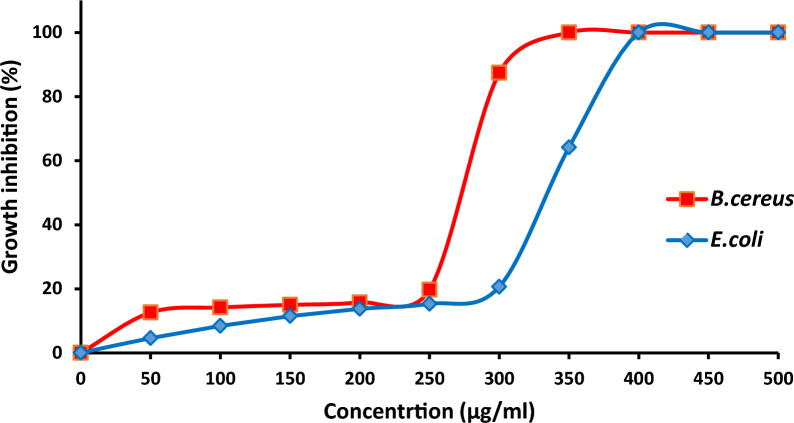
Growth inhibition percentage of *E. coli* and *B. cereus* in the presence of different concentrations of TPU/TiO_2_

The MIC of TPU/TiO_2_ against *B. cereus* and *E. coli* was determined. MIC is defined in vitro as the lowest concentration of TPU/TiO_2_ that causes complete inhibition of visible bacterial growth after incubation for 24 h. Figure 11 shows the growth inhibition curve of *E. coli* and *B. cereus* in the presence of different concentrations of TPU/TiO_2_. The antibacterial ratios of TPU/TiO_2_ MIC values against *B. cereus* and *E. coli* were 550 and 400 μg/mL, respectively. The MBC results were similar to MIC values which indicated the potent action of TPU/TiO_2_ against the tested bacteria. Similarly, Abdulazeem et al. [79] documented the MIC and MBC values of TiO_2_ against *E. coli* as 150 and 500 μg/mL, respectively. On the other Mahdy et al. reported that 40 μg/mL could inhibit *E. coli* growth as strong MIC [80]. While Younis et al. recorded that the MIC of TiO_2_ against *E. coli* reached 1500 μg/mL [81]

#### Ultrastructure research

**Fig. 12:**
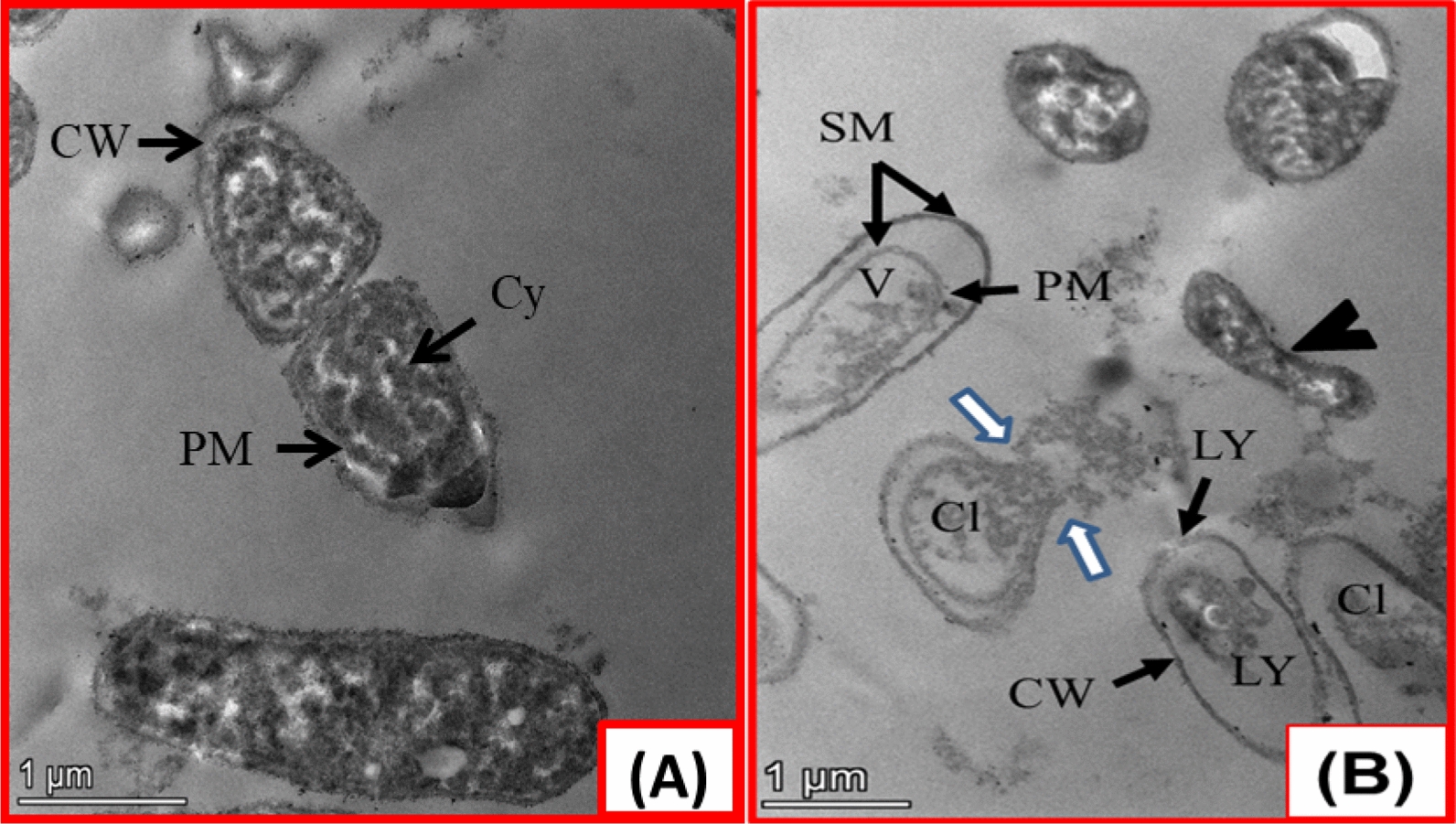
The bactericidal effect of TPU/TiO_2_ on the ultrastructure of *E. coli*. **a** A negative control (without TPU/TiO_2_). **b** A treated sample, there are malformed irregular rods (black arrowhead) with lysed cell walls (LY), separation between cell wall and plasma membrane (SM), departure of cellular content from the cell (white arrows), vacuole formation (V), and a complete cell lysis (Cl). CW, PM, and Cy refer to cell wall, plasma membrane and cytoplasm, respectively

TPU/TiO_2_'s antibacterial mechanism is unknown; however, TiO_2_NPs and thiourea may interact with microbial cell membranes and protein content to halt metabolic, respiratory, and cell division [82–84]. The ultrastructure morphology of untreated and TPU/TiO_2_-treated *E. coli* bacteria is shown in Fig. 12. Untreated *E. coli* had full cell walls and rod-like forms. The cell walls of *E. coli* looked wrinkled, broken, and detached from the plasma membrane, releasing cellular material. TPU/TiO_2_ also caused uneven rod deformity, vacuole development, and cell lysis. These studies suggest improving TPU/TiO_2_ for industrial and biological applications.

## Conclusion

We synthesized TiO_2_ from low-cost Ilmenite ore from Abu Ghalaga, Egypt. TiO_2_NPs and thiourea PUF formed TPU/TiO_2_, a novel nanocomposite. TPU/TiO_2_ was characterized using IR, UV–Vis, bandgap energy, magnetic susceptibility, chemical stability, and pH_PZC_. TPU/TiO_2_ inhibits *E. coli* and other microbial strains *e.g., B. cereus, A. niger.* TPU/TiO_2_ exhibits concentration-dependent cytotoxicity against MCF-7 and HepG-2 cells in vitro. Studying in vivo applications is intriguing.

### Supplementary Information

**Additional file 1: Table S1. **Cytotoxicity effects of TPU/TiO_2_ and Cisplatin (reference drug) against MCF-7 cells in vitro. **Table S2. **Cytotoxicity effects of TPU/TiO_2_ and Cisplatin (reference drug) against HepG-2 cells in vitro.

## Data Availability

The datasets used and/or analyzed during the current study are available from the corresponding author on reasonable request.

## Abbreviations

EDX: Energy-dispersive X-ray spectrometry
MTT: Methyl thiazolyl diphenyl-tetrazolium bromide
HepG-2: Hepatocellular carcinoma cell line
MCF-7: Michigan Cancer Foundation-7 (breast cancer cell line)
TPU: Thiourea polyurethane
TiO_2_NP: Titanium dioxide nanoparticles
PDT: Photodynamic treatment
PTKs: Protein tyrosine kinases
ROS: Reactive oxygen species
PUF: Polyurethane foam
OD: Optical density
HCC: Hepatocellular carcinoma
PURs: Polyester/ether urethanes
TEM: Transmission electron microscope
XRD: X-ray diffraction
IR: Infrared spectroscopy
IC_50_: The 50% inhibitory concentration
ANS: Arabian–Nubian Shield
EC_50_: The half-maximal effective concentration
S.D: Standard deviation
SI: Selective Index
