# Supplementary material for: Synthesis and biological evaluation of titanium dioxide/thiopolyurethane composite: anticancer and antibacterial effects
Source: BMC Chem. 2024 Feb 17;18(1):35. doi: 10.1186/s13065-024-01138-x (PMC10874576; doi:10.1186/s13065-024-01138-x)
Supplement: Supplementary file 1 — Additional file 1: Table S1. Cytotoxicity effects of TPU/TiO2 and Cisplatin (reference drug) against MCF-7 cells in vitro. Table S2. Cytotoxicity effects of TPU/TiO2 and Cisplatin (reference drug) against HepG-2 cells in vitro. [file 13065_2024_1138_MOESM1_ESM.docx]

**Table S1**

Cytotoxicity effects of TPU/TiO_2_ and Cisplatin (reference drug) against MCF-7 cells in vitro

| Concentration (µg/ml) | TPU/TiO_2_ | | Cisplatin |
| --- | --- | --- | --- |
|  | Viability % | Inhibitory% | Viability % |
| 0 | 100 | 0 | 100 |
| 3.9 | 100 | 0 | 52.85 |
| 7.8 | 100 | 0 | 46.71 |
| 15.6 | 100 | 0 | 34.62 |
| 31.25 | 99.72 | 0.28 | 23.79 |
| 62.5 | 88.16 | 11.84 | 14.68 |
| 125 | 61.29 | 38.71 | 7.83 |
| 250 | 42.93 | 57.07 | 4.98 |
| 500 | 17.45 | 82.55 | 3.72 |

**Table S2**

Cytotoxicity effects of TPU/TiO_2_ and Cisplatin (reference drug) against HepG-2 cells in vitro

| Concentration (µg/ml) | TPU/TiO_2_ | | Cisplatin |
| --- | --- | --- | --- |
|  | Viability % | Inhibitory% | Viability % |
| 0 | 100 | 0 | 100 |
| 3.9 | 100 | 0 | 47.89 |
| 7.8 | 100 | 0 | 40.62 |
| 15.6 | 98.46 | 1.54 | 31.87 |
| 31.25 | 92.31 | 7.69 | 22.98 |
| 62.5 | 78.64 | 21.36 | 12.39 |
| 125 | 49.05 | 50.95 | 6.75 |
| 250 | 26.78 | 73.22 | 4.31 |
| 500 | 8.21 | 91.79 | 3.02 |
